# Adolescents with ADHD and co‐occurring motor difficulties show a distinct pattern of maturation within the corticospinal tract from those without: A longitudinal fixel‐based study

**DOI:** 10.1002/hbm.26462

**Published:** 2023-08-22

**Authors:** Christian Hyde, Ian Fuelscher, Daryl Efron, Vicki A. Anderson, Tim J. Silk

**Affiliations:** ^1^ Centre for Social and Early Emotional Development, School of Psychology Deakin University Geelong Victoria Australia; ^2^ Department of Paediatrics University of Melbourne Melbourne Australia; ^3^ Murdoch Children's Research Institute Parkville Victoria Australia; ^4^ The Royal Children's Hospital Parkville Victoria Australia

**Keywords:** ADHD, corticospinal tract, fixel‐based analysis, motor function, white matter

## Abstract

It is well documented that attention‐deficit hyperactivity disorder (ADHD) often presents with co‐occurring motor difficulties. However, little is known about the biological mechanisms that explain compromised motor skills in approximately half of those with ADHD. To provide insight into the neurobiological basis of poor motor outcomes in ADHD, this study profiled the development of white matter organization within the cortico‐spinal tract (CST) in adolescents with ADHD with and without co‐occurring motor problems, as well as non‐ADHD control children with and without motor problems. Participants were 60 children aged 9–14 years, 27 with a history of ADHD and 33 controls. All underwent high‐angular resolution diffusion MRI data at up to three time points (115 in scans total). We screened for motor impairment in all participants at the third time point (≈14 years) using the Developmental Coordination Disorder Questionnaire (DCD‐Q). Following pre‐processing of diffusion MRI scans, fixel‐based analysis was performed, and the bilateral CST was delineated using TractSeg. Mean fiber density (FD) and fiber cross‐section (FC) were extracted for each tract at each time‐point. To investigate longitudinal trajectories of fiber development, linear mixed models were performed separately for the left and right CST, controlling for nuisance variables. To examine possible variations in fiber development between groups, we tested whether the inclusion of group and the interaction between age and group improved model fit. At ≈10 years, those with ADHD presented with lower FD within the bilateral CST relative to controls, irrespective of their prospective motor status. While these microstructural abnormalities persisted into adolescence for individuals with ADHD and co‐occurring motor problems, they resolved for those with ADHD alone. Divergent maturational pathways of motor networks (i.e., the CST) may, at least partly, explain motor problems individuals with ADHD.

## Graphical abstract

Children with ADHD showed atypical microstructure within the cortico‐spinal tract (CST) at 10 years, regardless of motor ability later in life. These CST abnormalities persisted into adolescence when ADHD co‐occurred with motor problems, yet resolved for those with ADHD alone. Maturation within motor networks may explain motor difficulties in ADHD.

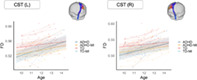

## 1 INTRODUCTION

Attention‐deficit hyperactivity disorder (ADHD) is a neurodevelopmental disorder characterized by inattention and/or impulsivity/hyperactivity. These symptoms lead to functional impairments in daily living, psychosocial disadvantage, and poorer academic performance (Sayal et al., 2018). Up to 50% of individuals with ADHD have co‐occurring motor difficulties (i.e., developmental coordination disorder [DCD]: Goulardins et al., 2015; Kaiser et al., 2015). Briefly, children with DCD present with motor skill levels significantly below that expected of their age, which impedes their ability to engage in activities of daily living that involve movement (e.g., dressing, eating, self‐care, using utensils, playing sport; Subara‐Zukic et al., 2022). The co‐occurrence of ADHD with reduced motor skill exacerbates the negative psychosocial impact of ADHD (Goulardins et al., 2015). However, little is known about the biological mechanisms underpinning motor difficulties in ADHD. Addressing this question is essential to clarifying whether the presentation of motor difficulties in children with ADHD reflects a characteristic feature of ADHD, or is distinct from the condition (see Goulardins et al., 2017).

Although motor development is subserved by a distributed neural network, maturation of the cortico‐spinal tracts (CST) during childhood is thought to be particularly important (Fuelscher, Hyde, Efron, et al., 2021). The CST is the major neural pathway for voluntary movement, with fibers emanating predominantly from the primary motor cortex to the spinal cord, where they synapse with lower motor neurons that control peripheral muscles (Welniarz et al., 2017). Given common reports of compromised motor skills in ADHD, it is perhaps not surprising that a growing body of research has reported atypical white matter organization within the CST in children with ADHD (Bu et al., 2019; Chuang et al., 2013; Fuelscher, Hyde, Anderson, et al., 2021; Hamilton et al., 2008; Hyde et al., 2021; van Ewijk et al., 2014). To date, few studies have directly investigated whether the differential profile of white matter organization within the CST might explain motor difficulties in those with ADHD. In one recent exception (Hyde et al., 2021), motor ability was assessed at the group level, since the study was unable to distinguish between those individuals with ADHD who did, and did not, present with poor motor outcomes. Given that CST microstructure differentiates otherwise healthy children with and without motor problems (Brown‐Lum et al., 2020; Dewey et al., 2019; Zwicker et al., 2012), it seems plausible that the profile of CST microstructure could differ between children with ADHD who present with, and without, co‐occurring motor problems.

Furthermore, the maturational profile of the CST might provide a window into the biological processes that explain why a substantial proportion of children with ADHD present with co‐occurring motor problems. Indeed, early disruption of the CST leads to poor motor outcomes in pediatric neurological disorders (e.g., as observed in cerebral palsy; Mailleux et al., 2020), with recent evidence also suggesting that motor skill training may partly reverse these microstructural impairments (Bleyenheuft et al., 2020). Similarly, movement rehabilitation in otherwise healthy children with low motor skill (i.e., DCD) has shown to induce plastic changes in microstructural properties within the CST (Izadi‐Najafabadi & Zwicker, 2021). This work suggests a longitudinal link between CST microstructure and motor outcomes in healthy and neurologically‐impaired pediatric groups. Given this, the presence of motor problems in those with ADHD may be preceded, at least in part, by atypical maturation of white matter properties within the CST. However, no study to date has longitudinally modeled CST white matter organization in those with ADHD, while simultaneously comparing the maturational profiles of those with typical motor function from those with co‐occurring motor difficulties.

To provide novel insight into the neurobiological basis of poor motor outcomes in ADHD, this study compared white matter maturation of the CST between children with ADHD who present with co‐occurring motor problems in adolescence and those who do not. We longitudinally modeled white matter organization of the bilateral CST in a large community‐based sample of children with and without ADHD aged 10–14 years (Silk et al., 2016). At the final timepoint (≈14 years), we were able to distinguish those with ADHD who presented with co‐occurring motor problems in adolescence from those who did not, using the Developmental Coordination Disorder Questionnaire (DCD‐Q: Wilson et al., 2007). Age‐matched children without ADHD were also included in the analysis as controls. These typically developing (TD) children were also screened for motor problems using the DCD‐Q, allowing us to distinguish CST development between neurotypical children with and without motor problems. White matter properties within the CST were modeled using fixel‐based analysis (FBA; Raffelt et al., 2017). Traditional tensor modeling (e.g., diffusion tensor imaging [DTI]) is unable to reconcile more than one fiber orientation within a given voxel, despite estimates that complex (i.e., multiple) fiber populations can be found in the majority (90%) of white matter voxels (Jeurissen et al., 2013). This not only compromises the biological plausibility of visual reconstructions of white matter tracts, but renders common tensor‐derived metrics (e.g., fractional anisotropy [FA]; mean diffusivity) difficult to interpret (Dhollander et al., 2021). FBA is a novel technique that provides fiber‐specific estimates of white matter organization. It does so via constrained spherical deconvolution (CSD), which is able to address the “crossing fiber” issue. Specifically, CSD modeling generates a fiber orientation distribution (FOD), which models specific fiber orientation within a voxel, even in the presence of multiple fiber populations. Unlike DTI methods, FBA is therefore able to generate metrics at the fiber population level, offering greater specificity and improving interpretability for voxel and tract level analyses. For our study, these included fiber density (FD), a microstructural measure of variation in density within a fiber population; and fiber cross section (FC), a macrostructural measure of the cross‐sectional region perpendicular to the fiber.

## 2 METHODS

**FIGURE 1:**
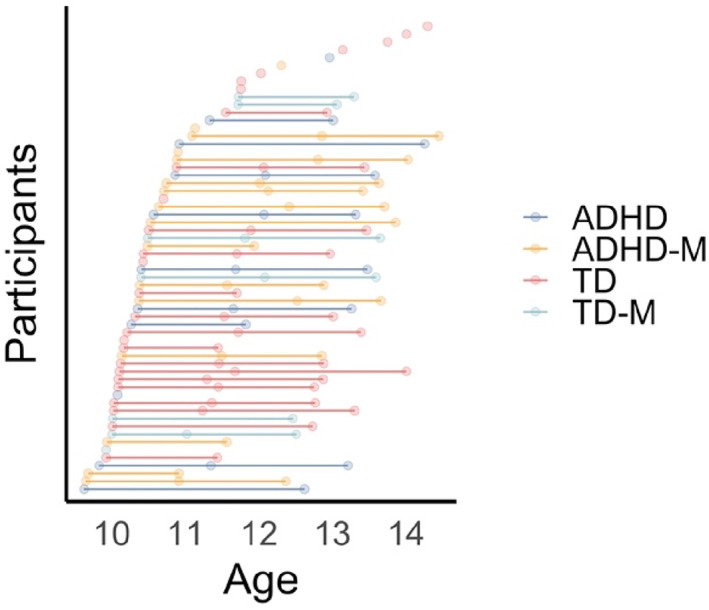
Distribution of imaging assessments by age and group. The final sample comprised a total of 132 observations (60 participants). This included 53 observations (26 participants) in the TD group, 16 observations (7 participants) in the TD + MI group, 25 observations (11 participants) in the ADHD group and 38 observations (16 participants) in the ADHD + MI group.

This study reports on a longitudinal community sample of 60 children aged between 9 and 14 years, 27 with and 33 controls without a history of ADHD (see Figure 1). The sample was recruited as part of the Children's Attention Project (CAP)/Neuroimaging Children's Attention Project (NICAP; Sciberras et al., 2013; Silk et al., 2016), a project approved by human research ethics committees at The Royal Children's Hospital (Melbourne, Australia) and Deakin University (Melbourne, Australia). Children were initially recruited at ages 6–8 years from 43 socio‐economically diverse primary schools in Melbourne, Australia. Following this, they participated in up to three neuroimaging assessments between the ages of 9 and 14 years. Neuroimaging assessments occurred at approximately 36 months (Wave 1: *M* = 10.31 years, *SD* = 0.36), 54 months (Wave 2: *M* = 11.72 years, *SD* = 0.44), and 72 months (Wave 3: *M* = 13.30 years, *SD* = 0.52) follow‐up. The total number of MRI scans included in the present set was 115. During the final wave of assessments (approximately 14 years of age), participants were screened for motor difficulties using the DCD‐Q (see below for details).

Intelligence was estimated at Wave 3 using the matrix reasoning sub‐test of the Wechsler Abbreviated Scale of Intelligence (WASI; Weschler, 2011). Participants socio‐economic status (SES) was determined using the Index of Relative Socio‐economic Advantage and Disadvantage (IRSAD), from the Socio‐economic Indexes for Areas (SEIFA), a relative measure of socio‐economic advantage/disadvantage based on 2011 Australian Census data (Australian Bureau of Statistics, 2011). The IRSAD (M = 1000, SD = 100) measures relative socio‐economic disadvantage, with lower scores indicating greater disadvantage. As per our earlier work (Fuelscher et al., 2023), children were identified as presenting with an internalizing disorder if they met criteria for depression, dysthymia, separation anxiety disorder, social phobia, generalized anxiety disorder, posttraumatic stress disorder, obsessive‐compulsive disorder, hypomania, or manic episode at any testing wave. Children were identified as presenting with an externalizing disorder if they met criteria for oppositional defiant disorder or conduct disorder at any testing wave. Since the sample was a community‐based sample, we did not identify or exclude children who were receiving a form of cognitive or motor intervention. Demographic and clinical characteristics for participants at Wave 3 are presented in Table 1.

**TABLE 1:** Clinical and demographic variables at Wave 3.

	Mean (SD)					Missing
	TD	TD + MI	ADHD	ADHD + MI	*p*	*n* (%)
*N* (%)	26 (43.3)	7 (11.7)	11 (18.3)	16 (26.7)		
Age, years	13.4 (0.5)	13.1 (0.5)	13.2 (0.4)	13.4 (0.7)	.451	
Male sex, *n* (%)	11 (42.3)	5 (71.4)	8 (72.7)	12 (75.0)	.111	
Left‐handed, *n* (%)	3 (11.5)	1 (14.3)	0 (0.0)	2 (12.5)	.672	
SES	1020.0 (45.0)	992.7 (72.6)	1036.0 (37.0)	1038.6 (33.6)	.146	2 (3.3)
DCDQ	72.7 (2.6)	55.0 (9.5)	72.2 (2.3)	54.2 (9.2)	**<.001** ^a^ ^,^ ^b^ ^,^ ^c^ ^,^ ^d^	
Conners 3 AI	0.4 (0.9)	0.7 (1.0)	3.7 (5.5)	9.4 (4.5)	**<.001** ^a^ ^,^ ^c^ ^,^ ^e^ ^,^ ^f^	1 (1.7)
Matrix reasoning	27.6 (1.9)	24.0 (9.2)	29.3 (1.9)	24.6 (4.0)	**.022**	14 (23.3)
Internalizing disorder (%)	2 (7.69)	0 (0.0)	2 (18.1)	4 (25.0)	**.**272	–
Externalizing disorder (%)	3 (11.54)	0 (0.0)	4 (36.36)	7 (43.75)	.**030** ^c^ ^,^ ^f^	–

*Note*: Typically Developing (TD), Motor Impaired (MI), Socioeconomic status was inferred using the Index of Relative Socio‐economic Advantage and Disadvantage (IRSAD), a measure of relative neighborhood socio‐economic disadvantage. Internalizing and externalizing disorders were assessed using the Diagnostic Interview Schedule for Children Version IV. Difference tests are based on ANOVA (continuous variables) or Chi‐squared tests (categorical variables). Values in bold indicate significant group differences (*p* < .05).

^a^ Post hoc analyses showed significant differences between the ADHD and ADHD + MI groups.

^b^ Post hoc analyses showed significant differences between the ADHD and TD + MI groups.

^c^ Post hoc analyses showed significant differences between the ADHD + MI and TD groups.

^d^ Post hoc analyses showed significant differences between the TD + MI and TD groups.

^e^ Post hoc analyses demonstrated significant differences between the ADHD and TD groups.

^f^ Post hoc analyses showed significant differences between the ADHD + MI and TD + MI groups.

### 2.1 Clinical assessment

#### 2.1.1 ADHD status

Screening for ADHD diagnosis occurred at recruitment, and was repeated at Waves 1 and 3, using the parent and teacher Conners 3 ADHD Index (Conners, 2008). The NIMH Diagnostic Interview Schedule for Children IV (DISC‐IV; Shaffer et al., 2000) was administered to confirm ADHD status. Conversely, non‐ADHD controls screened negative for ADHD by both parent and teachers, and did not meet diagnostic criteria on the DISC‐IV. We included participants in our ADHD group if they met criteria for the disorder at any of the three testing waves. This meant that a portion of participants persistently met ADHD criteria across timepoints (59%), while others met criteria at at least one (but not all) time points (41%). Limited sample size precluded us from conducting separate analysis to determine whether the effects of interest (i.e., CST development) differed between these two groups of children with ADHD. However, preliminary sensitivity analyses failed to detect a difference in ADHD symptoms rating (as measured by the Conners 3 ADHD Index [CAI]) between those who showed persistent ADHD symptoms across waves, and those that met ADHD criteria at at least one time point (but not all) ‐ see Supporting Information A.

#### 2.1.2 Motor outcomes

Children at Wave 3 were also administered the DCD‐Q (Wilson et al., 2007), a well‐validated parent‐report measure for the identification of motor difficulties. The 15‐item Likert‐scale questionnaire assesses a child's ability to engage in everyday activities involving movement, measuring motor ability across three domains “control during movement”, “fine motor and handwriting/printing”, and “general coordination”. A total score is generated by aggregating scores across all items, with lower scores indicating greater motor impairment. In the absence of Australian psychometric data for the DCD‐Q, we adopted revised cut‐off scores (see also Cairney et al., 2019) for the DCD‐Q based on the 95% confidence intervals of the DCD‐Q Total score for the non‐ADHD participants in this sample (65.87–72.07). Children who scored below the lower range (i.e., 65 or below) were assigned to our DCD group. Using our revised cut‐off, we observed that over half (59%) of our sample of individuals with ADHD presented with co‐occurring motor difficulties, consistent with prior prevalence estimates of ≈50% (Goulardins et al., 2015). The similarity in prevalence of motor problems observed in those with ADHD in this study and earlier work provides support for the revised cut‐off scores that we adopted for the DCD‐Q in our sample of Australian children. Furthermore, as seen in Table 1, the mean DCD‐Q score for the ADHD + DCD and DCD group fell comfortably below the suggested cut‐off score of 57 for indicative DCD for 10–14 year olds (age range of study participants at Wave 3). Nonetheless, where participants met the above criteria for DCD in our study, we opted to refer to this group as “*motor impaired*” (MI), rather than as having DCD.

#### 2.1.3 Exclusion criteria

Those children who were currently medicated, or had a known history of ADHD medication use, were excluded from the sample since ADHD medication may impact motor function (Kaiser et al., 2015). Additional exclusion criteria included intellectual disability, severe medical conditions, genetic disorders, moderate–severe sensory impairment and neurological problems. Furthermore, where parents had insufficient English skills to complete the interviews or questionnaires, children were excluded. Finally, those children who were not compatible with the MRI (for any reason) were excluded.

### 2.2 MRI acquisition and processing

Diffusion and T1‐weighted images were acquired using a 3T Siemens scanner across all three time‐points. While Waves 1 and 2 were acquired on the Tim Trio scanning system, following an upgrade, Wave 3 was captured on a the MAGNETOM Prisma system. As a result, scanner was included as a nuisance covariate in our statistical modeling. High‐angular resolution diffusion imaging (HARDI) data were acquired in the transverse plane with an anterior–posterior phase encoding direction. Sixty gradient directions, b‐value = 2800 s/mm^2^ and four interleaved b = 0 volumes were acquired (TR = 3200 ms, TE = 110 ms, echo spacing = 0.69 ms, FOV = 260 mm, multi‐band factor = 3 and 2.4 mm isotropic voxels). A reverse phase‐encoded image was acquired to correct for magnetic susceptibility‐induced distortions during EPI acquisition. High resolution T1‐weighted, multi‐echo MPRAGE images were acquired in the sagittal plane with in‐scanner motion correction (TR = 2530 ms, TE = 1.77, 3.51, 5.32, 7.2 ms, flip angle 7°, voxel size = 0.9 mm^3^).

Diffusion data were pre‐processed using the MRtrix3 software package using the recommended FBA pipeline (Tournier et al., 2019). This included denoising, motion, eddy current and susceptibility distortion correction, and bias field correction. For details regarding quality control and MRI data exclusion, please see Supporting Information B. Following pre‐processing, data were upsampled to an isotropic voxel size of 1.30 mm^3^. Group average response functions for gray matter, white matter, and CSF used to generate individual FOD maps using single‐shell three‐tissue constrained spherical deconvolution (SS3T‐CSD; Dhollander et al., 2016). Next, FOD images underwent intensity normalization to ensure that FOD magnitudes were consistent across participants (Raffelt et al., 2017). A FOD template was generated based on a representative subset of 24 participants (12 ADHD, 12 non‐ADHD) matched on age and sex from the larger NICAP cohort (see Thomson et al., 2022). Fixel metrics including FD and FC (log) were calculated for each participant across white matter fixels. FC (log) was calculated rather than FC to ensure normal distribution of the variable.

The left and right CST were delineated using the automated TractSeg method (Wasserthal et al., 2018) in the population FOD template to segment those voxels that corresponded to the bilateral CST in each individual. TractSeg was adopted since it offers a balance between the accuracy of manual delineation and the reliability of atlas‐based approaches (Genc et al., 2020). In doing so, TractSeg offers an advantage over commonly adopted voxel‐based analyses (including tract‐based spatial statistics [TBSS]). For example, TBSS involves placing a tract‐skeleton/template over the purported middle of white matter tracts, then generating DTI based metrics (e.g., FA; Smith et al., 2006). Aside from the previously mentioned limitations of DTI derived metrics, it is now well documented that these templates may not represent the “centre” of tracts, as suggested, which limits anatomical accuracy (Bach et al., 2014). Conversely, TractSeg is applied directly to the individual study FODs where machine learning is applied to delineate tracts via CSD, meaning that the tracts generated more closely align with structural neuroanatomy.

**FIGURE 2:**
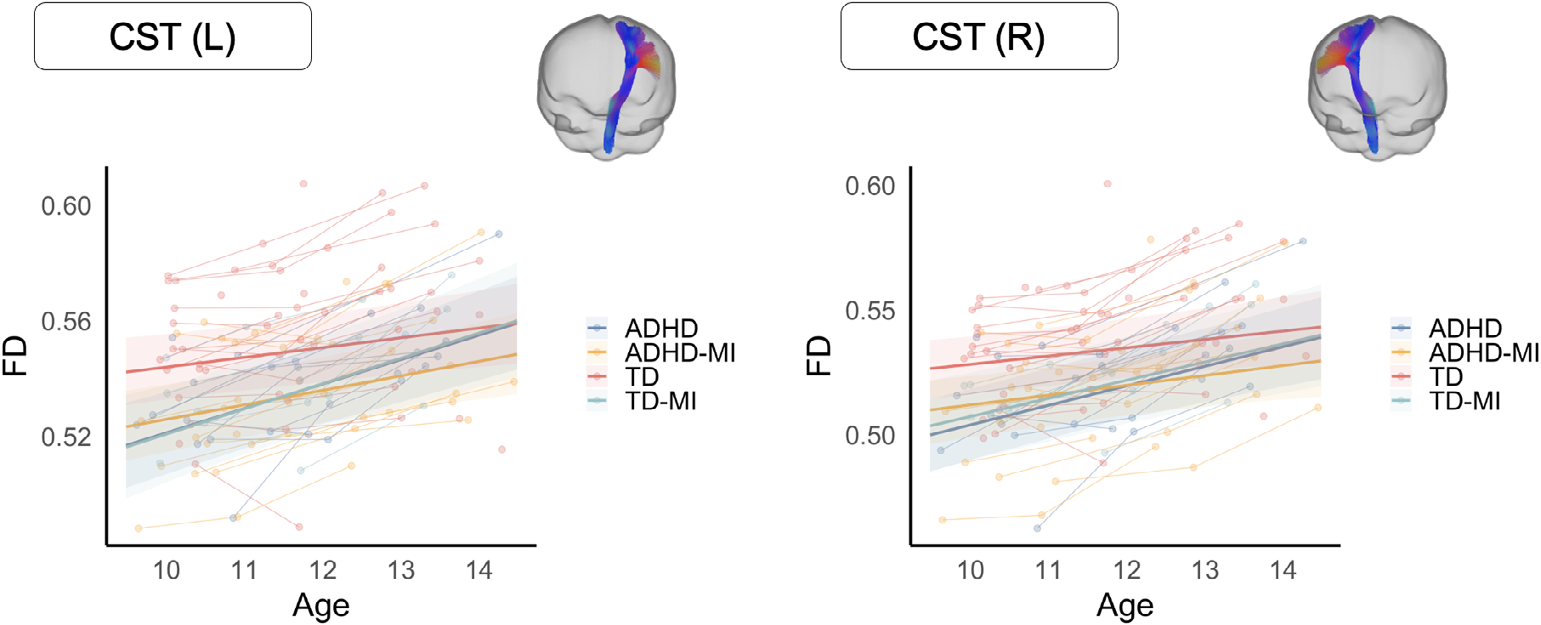
Longitudinal mixed linear models predicting fiber density (FD). Tracts showing age × group interaction effects (*p* < .05). Colored areas represent the 95% confidence interval. CST, corticospinal tract; L, left; R, right.

In our study, tract orientation maps were generated for the left and right CST, from which probabilistic tractography was conducted with 10,000 streamlines. The resultant CST tractograms were then converted to fixel maps, which were used to crop those fixels that correspond to the streamlines generated for the left and right CST (see Figure 2). Fixel metrics including FD and FC were then extracted for each participant for the left and right CST, and the mean was taken for each participant at each time point.

### 2.3 Design and analysis

To investigate longitudinal trajectories of fiber development, linear mixed models were performed in R using the lmer and lme functions of the lme4 and nlme packages (Bates et al., 2014; Pinheiro et al., 2017; R Core Team, 2013). Probability values for model parameters were obtained using the lmerTest package (Kuznetsova et al., 2017). Models were run separately for the left and right CST. Analyses were adjusted for sex, scanner upgrade, and head motion. As per the recommended FBA pipeline (Dhollander et al., 2021), analyses involving FC were conducted using log(FC).

Analyses first compared null (1), linear (2), and quadratic (3) models to identify the most parsimonious developmental model for the left and right CST (Models 1–3; Table 2). A more complex model was chosen if likelihood ratio tests indicated that the model differed significantly from the simpler model (*p* < .05; Lewis et al., 2011) and the Akaike information criterion (AIC) indicated better model fit (ΔAIC > 2) (Vrieze, 2012). Models were conducted with a random intercept (d_i_) to account for repeated observations per participant.

**TABLE 2:** Linear mixed models assessing longitudinal fiber development.

Model	Equation
1	Fiber metric ~ intercept + d_i_ + β_1_ (sex) + β_2_ (scanner) + β_3_ (motion)
2	Fiber metric ~ intercept + d_i_ + β_1_ (sex) + β_2_ (scanner) + β_3_ (motion) + β_4_ (age)
3	Fiber metric ~ intercept + d_i_ + β_1_ (sex) + β_2_ (scanner) + β_3_ (motion) + β_4_ (age) + β_5_ (age^2^)
4	Fiber metric ~ intercept + d_i_ + β_1_ (sex) + β_2_ (scanner) + β_3_ (motion) + β_4_ (age) + β_5_ (group)
5	Fiber metric ~ intercept + d_i_ + β_1_ (sex) + β_2_ (scanner) + β_3_ (motion) + β_4_ (age) + β_5_ (group) + β_6_ (age × group)

*Note*: d_i_ = random intercept for each participant; β_n_ = regression parameter.

To examine possible variations in fiber development between the TD children, typically developing children with MI (TD + MI), ADHD, and ADHD + MI groups, analyses tested whether the inclusion of group and the interaction between age and group improved model fit beyond the best fitting developmental model (Models 4 and 5; Table 2). Again, a more complex model was selected based on likelihood ratio tests and lower AIC.

## 3 RESULTS

### 3.1 Demographic and clinical characteristics

As shown in Table 1, the groups did not differ in age, handedness, SES, or matrix reasoning at Wave 3. Group differences were observed for Conners 3 ADHD Index (CAI) and matrix reasoning scores.

### 3.2 Variations in fiber development

Consistent with earlier work (Lebel et al., 2019), both white matter microstructure (FD) and morphology (FC) of the CST increased in a linear fashion over development. Full model details are presented in the Supporting Information C).

#### 3.2.1 White matter microstructure (FD)

For both the left (*p* = .002) and right (*p* = .003) CST, the interaction model (5) demonstrated the best model fit. As shown in Figure 2, the interaction effect suggested that the ADHD and TD + MI groups had lower FD (relative to the TD group) around age 10 but showed accelerated microscopic fiber development between the ages of 10 and 14 years. Analysis further demonstrated that the ADHD + MI group had lower FD than the TD group in both the left (*p* = .039) and right (*p* = .046) CST across the study period. The rate of fiber development did not differ between the ADHD + MI and TD groups (see Figure 2). Full model details are presented in the Supporting Information B.

#### 3.2.2 White matter morphology (FC)

No significant differences and/or variations in fiber development were observed between the groups. Full model details are presented in the Supporting Information.

## 4 DISCUSSION

This study aimed to investigate whether the maturational profile of the CST could provide insight into the neurobiological basis of co‐occurring motor difficulties in children with ADHD. We observed that, during mid‐childhood (10 years), those with ADHD presented with lower FD within the bilateral CST relative to those without ADHD, regardless of their prospective motor status (i.e., ADHD + MI and ADHD). These microstructural abnormalities persisted for those individuals who presented with co‐occurring motor problems at adolescence (ADHD + MI). Conversely, the atypical CST microstructure observed in children with ADHD resolved in those individuals who presented with typical motor skill levels at adolescence. Divergence in the maturational profile of the CST between those with ADHD + MI and ADHD supports the view that atypical development of motor networks may explain, at least partly, the presence of motor difficulties in a substantial sub‐set of those with ADHD. It also lends weight to argument that poorer motor skill in ADHD is distinct from the core ADHD symptom profile.

In‐keeping with earlier accounts (Bu et al., 2019; Chuang et al., 2013; Fuelscher, Hyde, Anderson, et al., 2021; Hamilton et al., 2008; Hyde et al., 2021; van Ewijk et al., 2014), we observed that children with ADHD presented with atypical white matter microstructure within the CST. This was shown by lower FD in the bilateral CST relative to non‐ADHD controls. FD is thought to reflect the number, or density, or axons within a voxel (Genc et al., 2018; Raffelt et al., 2017). Our results may reflect a lower number, or diameter, of axons in the CST for those with ADHD. We extended earlier work by distinguishing between those children with ADHD who presented with motor difficulties in later childhood (ADHD + MI), from those who did not (ADHD). Interestingly, at 10 years of age, both groups of children with ADHD (i.e., ADHD + MI and ADHD) presented with similarly lower FD in the bilateral CST. This same effect was also observed in TD children with motor problems (TD + MI). Our results are partially consistent with functional accounts of the CST, which is thought to be primarily involved in the expression of voluntary movements (Welniarz et al., 2017). However, the presence of atypical CST microstructure in 10 year olds with ADHD who had typical motor function at adolescence indicates that abnormal CST microstructure may have implications for children with ADHD beyond the motor realm. This view is supported by recent work demonstrating that increased FD of the CST in adolescence‐early adulthood is associated with a greater reduction in hyperactivity/impulsivity symptom expression across the preceding 3–4 years (Damatac et al., 2022). Taken together, this work suggests that CST microstructure may play a role in symptom expression in children with ADHD, irrespective of motor status.

With respect to the maturational profile of microstructure within the CST, we observed distinct linear profiles for children with ADHD + MI and those with ADHD alone. This was demonstrated by a significant group by age interaction effect for FD of the bilateral CST, with the linear effect offering the most parsimonious model fit. The latter is consistent with longitudinal accounts of CST maturation, which often show protracted linear‐like maturation of white matter organization within the CST from childhood through the teen years and beyond (see Lebel et al., 2019). However, we observed consistently lower FD in the bilateral CST for those adolescents with ADHD + MI relative to our TD controls across the preceding 4‐year developmental period. In contrast, while adolescents with ADHD initially presented with lower FD of the CST at 10 years, by adolescence (i.e., 14 years) their CST microstructure had largely normalized, presenting with similar FD of the CST to TD controls. This same pattern was also observed in those control adolescents who presented with motor problems at 14 years (TD + MI). The latter effect is interesting, since unlike those with ADHD + MI, the presence of motor problems alone in adolescence was not precipitated by consistent CST abnormalities between 10 and 14 years. There is preliminary evidence that microstructure within the CST may improve over a relatively short period (3 months) in otherwise TD children with motor problems (i.e., DCD), where no such effect is observed in those with co‐occurring ADHD + DCD (Izadi‐Najafabadi & Zwicker, 2021). This data is consistent with the present findings, which indicate that even relative to those with MI alone, atypical CST microstructure may be more developmentally persistent in those with combined ADHD + MI. Taken together, we observed that while all three non‐control groups (ADHD, ADHD + MI, TD + MI) showed lower FD of the bilateral CST in mid‐childhood (i.e., 10 years), this effect only persisted for the ADHD + MI group, having spontaneously dissipated by adolescence in the ADHD and TD‐M groups.

Our data indicate that the maturational profile of sensorimotor microstructure (more specifically, the bilateral CST) from late childhood into adolescence, may offer insight into the presence of motor difficulties in a sub‐set of adolescents with ADHD. Though recent work has mapped the development of microstructure of the CST in those with ADHD (Chiang et al., 2023), it did not evaluate its impact on functional motor outcomes. Thus, the degree to which microstructure of the CST, and its maturational profile, might distinguish those with ADHD who present with motor problems from those who do not was unclear. Our study is the first to demonstrate that while atypical microstructure of the CST in childhood may be a common trait of ADHD (regardless of motor outcomes later in life), the persistence of microstructural abnormalities into adolescence is unique to those who present with poorer motor outcomes in adolescence.

The divergence in maturational pathways of the CST between those with ADHD + MI and ADHD supports recent arguments that motor difficulties observed in ADHD reflect a distinct symptom cluster rather than a core phenotypic characteristic of ADHD (Farran et al., 2020; Goulardins et al., 2015; Lee et al., 2021). This is in‐keeping with a small existing body of literature that suggests distinct (yet overlapping) neural profiles for children with ADHD + DCD, relative to either disorder in isolation (Izadi‐Najafabadi & Zwicker, 2021; Langevin et al., 2015). For example, recent work has demonstrated that children with DCD show plastic microstructural changes in sensori‐motor tracts such as the CST following a 10‐week *Cognitive Orientation to Occupational Performance* (CO‐OP) intervention, an effect not observed in those with DCD + ADHD (Izadi‐Najafabadi & Zwicker, 2021). This study highlights that the CST may not be as susceptible to experientially driven plasticity in those with combined ADHD + MI relative to those with just one of the two disorders in isolation.

If the motor difficulties observed in ADHD reflect a distinct symptom cluster rather than a core characteristic of ADHD, this has several important clinical implications. First, it supports recent calls for the need for early assessment of motor skills in children with ADHD (and vice versa), and direct intervention for motor symptoms (Blank et al., 2019; Lange, 2018). Second, it indicates that standard treatments that address core ADHD symptoms may have limited impact on the motor symptoms of those with co‐occurring ADHD and DCD. This view is supported by meta‐analysis showing that motor intervention provides the most promising treatment effects for improving motor skill relative to non‐motor interventions (including medication; Kleeren et al., 2023).

Our data also showed that inattentive symptoms were significantly higher in the ADHD + MI group, relative to the ADHD group at 14 years (i.e., Wave 3). This raises the question as to whether greater inattention in the ADHD + MI group might explain the differential profile of CST maturation observed in this group relative to the ADHD group, rather than MI. We note that were inattention the primary driver of CST microstructural abnormalities in those with ADHD + MI, then it would be expected that the ADHD group would have also maintained some degree of CST abnormality relative to the TD group across development, since they too experienced elevated inattention relative to controls. This was not the case. We can therefore be confident that inattention does not, at least entirely, explain the divergent CST maturational profile of those with ADHD + MI and ADHD.

We acknowledge several limitations of the present study, which warrant consideration. We only assessed motor outcomes in our sample once they reached adolescence (Wave 3). Without motor assessments in earlier waves of assessment, we are unable to verify the motor status of our sample in childhood. Even still, our data are the first to demonstrate that atypical development of the CST (or indeed, persistently lower FD) from late childhood to adolescence presages MI in those with adolescent ADHD. In doing so, it provides novel insight into the role of maturation within the CST in motor outcomes in ADHD. Future work should monitor motor status in those with ADHD longitudinally in order to determine whether motor status fluctuations across childhood might impact the contribution of CST microstructure to motor outcomes in those with ADHD. Finally, while the DCD‐Q is often used to screen for the presence of motor difficulties, we note that a formal diagnosis of DCD would typically require a follow‐up assessment including a standardized battery of movement function (e.g., Movement Assessment Battery for Children—second edition [MABC‐2]; Henderson et al., 2007). The latter was not feasible within the scope of the broader CAP project, which involved deep phenotyping of participants and detailed clinical, standardized, and experimental assessment (see Silk et al., 2016 for a full description of the CAP procedure). The initial validation data suggest that the DCD‐Q has a reported sensitivity and specificity of 85% and 71%, respectively (Wilson et al., 2009). Furthermore, recent work has highlighted very high sensitivity and negative predictive validity of the DCD‐Q when referenced to a formal diagnosis of DCD in children with co‐occurring neurodevelopmental disorders (Van Damme et al., 2022). For this reason, the DCD‐Q is commonly used to screen for motor problems in children with other neurodevelopmental disorders, such as ADHD and ASD (Bhat, 2022; Ketcheson et al., 2021; Shaw et al., 2016). Still, future work should consider whether the profile of CST maturation observed in those with ADHD and ADHD + MI in the current study holds where DCD is confirmed in the latter group using a standardized battery of motor function.

Finally, participants were included in our ADHD group if they had a history of ADHD, meaning that a portion of participants persistently met ADHD criteria across timepoints, while others met criteria at at least one (but not all) time points (see Section 2 for details). Though it would have been interesting to investigate whether CST development differed between these two groups, our sample size prevented us from doing so, which stands as a limitations of the study. However, our decision to prospectively group participants from recruitment (based on a childhood history of ADHD) is in‐keeping with recent arguments to examine ADHD dimensionally rather than as discrete categories (Levy et al., 1997; McLennan, 2016; Wu et al., 2017). Indeed, even those children who no longer meet diagnostic criteria for ADHD often present with functional impairments similar to those with a persistent diagnosis of ADHD. In support, sensitivity analyses in our own study here failed to detect a difference in ADHD symptoms rating between those who showed persistent ADHD symptoms across waves, and those that met ADHD criteria at at least one time point (but not all)‐ see Supporting Information A. Nonetheless, our findings should be interpreted in the context of the complex and variable course of ADHD, and future work would benefit from investigating whether the effects observed in our study alter as a function of the persistence (or remittance) of ADHD diagnosis. Similarly, as a general note, while our sample size was strong for a longitudinal neuroimaging study in ADHD, it remains statistically modest. Our findings should also be considered in the context of this limitation.

In conclusion, we observed divergent maturational pathways in CST microstructure between adolescents with ADHD who did (ADHD + MI), and did not (ADHD), present with co‐occurring motor difficulties. More specifically, while those adolescents with ADHD + MI showed persistent reductions in CST microstructure between 10 and 14 years, those with ADHD alone “caught up” to their neurotypical counterparts across the same time period, an effect also observed in non‐ADHD adolescents who presented with low motor ability. We argue that the maturational profile of the CST may provide a window into the mechanisms by which many children with ADHD present with co‐occurring motor problems.

## FUNDING INFORMATION

This project is funded by Project Grants from the Australian National Health and Medical Research Council (NHMRC) (1065895, 1008522).The research was supported by the Murdoch Children’s Research Institute, the Royal Children’s Hospital, the Royal Children’s Hospital Foundation, the Department of Paediatrics at the University of Melbourne, and the Victorian Government’s Operational Infrastructure Support Program.

## Supporting information


**Data S1:** Supporting Information.

## Data Availability

The data that support the findings of this study are available on request from the corresponding author. The data are not publicly available due to privacy or ethical restrictions.
