# Supplementary material for: Adolescents with ADHD and co‐occurring motor difficulties show a distinct pattern of maturation within the corticospinal tract from those without: A longitudinal fixel‐based study
Source: Hum Brain Mapp. 2023 Aug 22;44(16):5504–13. doi: 10.1002/hbm.26462 (PMC10543105; doi:10.1002/hbm.26462)
Supplement: Supplementary file 1 — Data S1: Supporting Information. [file HBM-44-5504-s001.docx]

**Supplementary materials A**

A one-way ANOVA was conducted to compare mean CAI scores across groups (ADHD _persistent_, ADHD_remitted_ and TD). Analyses revealed a significant main effect for group, *F* (2, 56)= 22.17, *p* <.001, η^2^*_p_* = .44. Tukeys post-hoc tests demonstrated that the ADHD_Persistent_ group (*M*= 7.56, *SD*= 5.91) showed significant greater mean CAI than the TD group (*M*= .438, *SD*= 0.91), t(56)= -6.06, *p_Tukey_* < .001, *d* = -1.86. Similarly, the ADHD _remitted_ group (*M*= 6.36, *SD*= 5.26) showed significant greater mean CAI than the TD group t(56)= -4.41, *p_Tukey_* < .001, *d* = -1.54. No group differences was observed between the mean CAI of the ADHD_Persistent_ and ADHD _remitted_ groups respectively, t(56)= -0.797, *p_Tukey_* = .707, *d* = 0.31.

**Supplementary materials B**

**Data exclusion**

The scans included in this study were captured as part of a larger study, and pre-processing and quality control occurred according to the steps reported in our recent work (Fuelscher et al., 2023). To summarize, neuroimaging data was acquired from 471 scans. Following processing, quality control protocols, and exclusions according to the presence/absence of DCD-Q data at Wave 3, the final sample comprised 115 scans (see Supplementary Figure S1 for a workflow).

**Head motion**

To correct for the effects of head motion, we adopted a novel pre-processing approach using FSL, which included including slice-to-volume correction and slice-wise outlier replacement (Andersson et al., 2017; Andersson, Graham, Zsoldos, & Sotiropoulos, 2016). We excluded scans with >10% outlier volumes (defined as > 10% outlier slices). Scans beyond three times the interquartile range in absolute restricted motion or relative restricted motion were also excluded. Finally, we calculated the number of dropout slices for each participant using the available eddy QC metrics, and included this variable as a covariate in all analyses (see also Dimond et al., 2020; Fuelscher, Hyde, Anderson, & Silk, 2021),


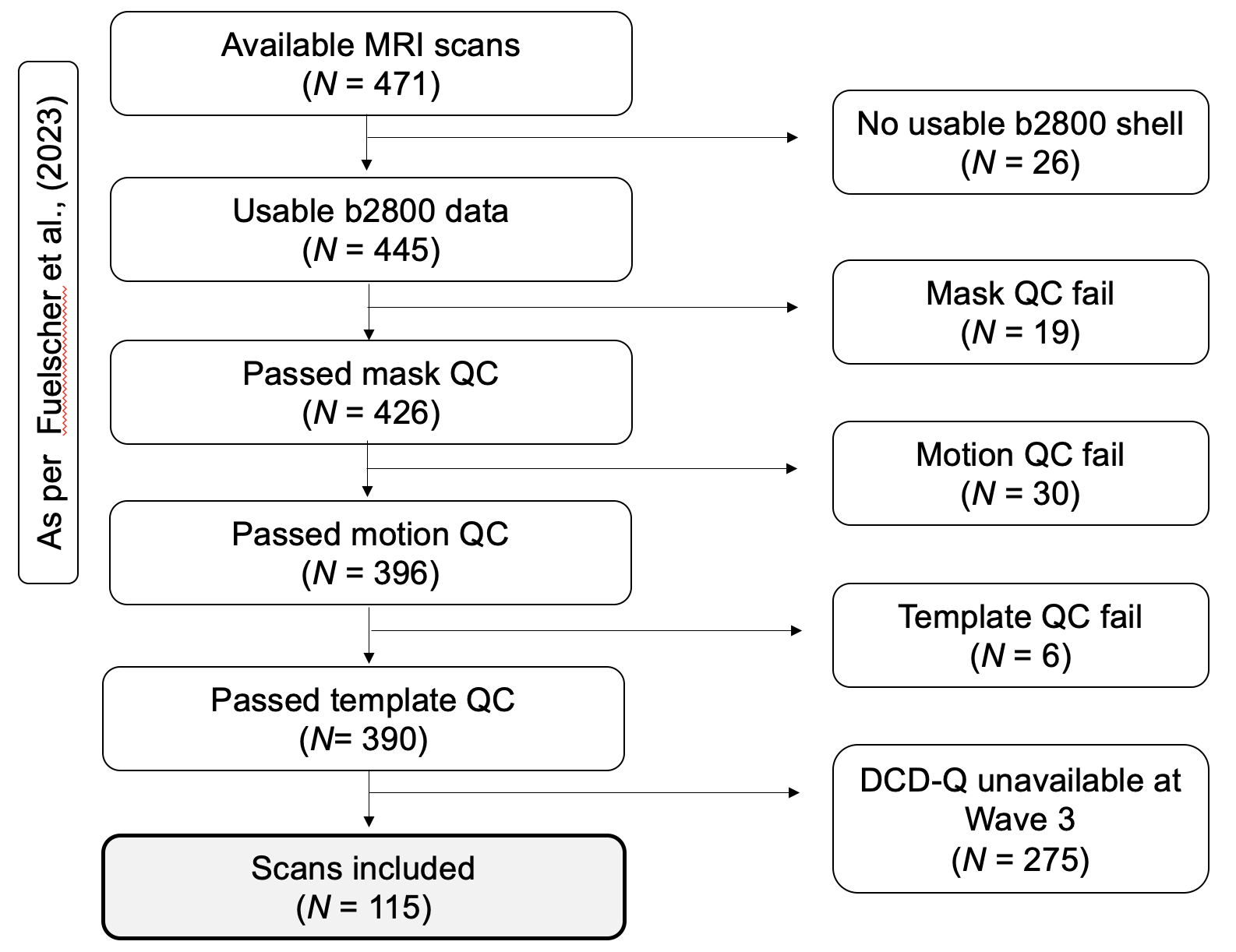


**Supplementary Figure S1.** Diffusion MRI exclusions following quality control (QC) (these are also reported in Fuelscher et al., 2023). Individual subject masks were created from the intersection of the anatomical [based on T1 weighted images derived using FreeSurfer (Fischl, 2012)] and diffusion masks [based on the diffusion weighted images derived using MRtrix3 (Tournier et al., 2019)]. Mask QC involved checking that subject masks included those regions of the brain intended for analysis. Template QC involved checking that subject masks (registered to the population template) included those regions of the brain intended for analysis. Of those scans remaining, those which corresponded to a participant with DCD-Q data available at Wave 3 were included.

**Supplementary materials B References**

Andersson, J. L. R., Graham, M. S., Drobnjak, I., Zhang, H., Filippini, N., & Bastiani, M. (2017). Towards a comprehensive framework for movement and distortion correction of diffusion MR images: Within volume movement. *Neuroimage, 152*, 450-466 %@ 1053-8119.

Andersson, J. L. R., Graham, M. S., Zsoldos, E., & Sotiropoulos, S. N. (2016). Incorporating outlier detection and replacement into a non-parametric framework for movement and distortion correction of diffusion MR images. *Neuroimage, 141*, 556-572 %@ 1053-8119.

Dimond, D., Rohr, C. S., Smith, R. E., Dhollander, T., Cho, I., Lebel, C., . . . Bray, S. (2020). Early childhood development of white matter fiber density and morphology. *Neuroimage*, 116552.

Fischl, B. (2012). FreeSurfer. *Neuroimage, 62*(2), 774-781 %@ 1053-8119.

Fuelscher, I., Hyde, C., Anderson, V., & Silk, T. J. (2021). White matter tract signatures of fiber density and morphology in ADHD. *Cortex, 138*, 329-340.

Fuelscher, I., Hyde, C., Thomson, P., Vijayakumar, N., Sciberras, E., Efron, D., . . . Silk, T. J. (2023). Longitudinal Trajectories of White Matter Development in Attention-Deficit/Hyperactivity Disorder. *Biological Psychiatry: Cognitive Neuroscience and Neuroimaging %@ 2451-9022*.

Tournier, J.-D., Smith, R., Raffelt, D., Tabbara, R., Dhollander, T., Pietsch, M., . . . Connelly, A. (2019). MRtrix3: A fast, flexible and open software framework for medical image processing and visualisation. *Neuroimage*, 116137.

**Supplementary Material C: Table SC.** Model comparisons for linear mixed models assessing longitudinal fiber development

|  |  | FD | | | |
| --- | --- | --- | --- | --- | --- |
|  | Model | AIC | logLik | Chisq | *p* |
| CST left | 1 | -711.79 | 361.90 |  |  |
|  | 2 | **-726.37** | **370.19** | **16.58** | **< .001** |
|  | 3 | -724.49 | 370.24 | 0.12 | 0.731 |
|  | 4 | -725.67 | 372.84 | 5.30 | 0.151 |
|  | 5 | **-734.28** | **380.14** | **14.61** | **0.002** |
| CST right | 1 | -723.53 | 367.76 |  |  |
|  | 2 | **-737.65** | **375.82** | **16.12** | **< .001** |
|  | 3 | -735.75 | 375.87 | 0.10 | 0.752 |
|  | 4 | -737.42 | 378.71 | 5.77 | 0.123 |
|  | 5 | **-745.27** | **385.63** | **13.85** | **0.003** |

*Note.* Values in bold indicate the best fitting developmental model (models 1-3) and the best fitting ADHD model (models 4-5). Developmental models examined whether age (or the quadratic effect of age) improved model fit beyond a null model with covariates only. ADHD models examined whether the inclusion of group (TD, TD+MI, ADHD, ADHD+MI) and the interaction between age and group improved model fit beyond the best fitting developmental model. CST = corticospinal tract; FD = fiber density; AIC = Akaike information criterion; logLik = log-likelihood; Chisq = Chi-square.
